# Associations between fluid overload and outcomes in critically ill patients with acute kidney injury: a retrospective observational study

**DOI:** 10.1038/s41598-023-44778-0

**Published:** 2023-10-13

**Authors:** Yosuke Hayashi, Takashi Shimazui, Keisuke Tomita, Tadanaga Shimada, Rie E. Miura, Taka-aki Nakada

**Affiliations:** 1 Department of Emergency and Critical Care Medicine, Chiba University Graduate School of Medicine, 1-8-1 Inohana, Chuo, Chiba, 260-8677 Japan; 2 Smart119 Inc., 2-5-1 Chuo, Chiba, 260-0013 Japan

**Keywords:** Kidney, Risk factors

## Abstract

Increased fluid overload (FO) is associated with poor outcomes in critically ill patients, especially in acute kidney injury (AKI). However, the exact timing from when FO influences outcomes remains unclear. We retrospectively screened intensive care unit (ICU) admitted patients with AKI between January 2011 and December 2015. Logistic or linear regression analyses were performed to determine when hourly %FO was significant on 90-day in-hospital mortality (primary outcome) or ventilator-free days (VFDs). In total, 1120 patients were enrolled in this study. Univariate analysis showed that a higher %FO was significantly associated with higher mortality from the first hour of ICU admission (odds ratio 1.34, 95% confidence interval 1.15–1.56, *P* < 0.001), whereas multivariate analysis adjusted with age, sex, APACHE II score, and sepsis etiology showed the association was significant from the 27th hour. Both univariate and multivariate analyses showed that a higher %FO was significantly associated with shorter VFDs from the 1st hour. The significant associations were retained during all following observation periods after they showed significance. In patients with AKI, a higher %FO was associated with higher mortality and shorter VFDs from the early phase after ICU admission. FO should be administered with a physiological target or goal in place from the initial phase of critical illness.

## Introduction

Fluid resuscitation is essential during initial shock management in critically ill patients who have vasodilation disorder and/or hypovolemia^1,2^. Along with fluid resuscitation, excessive fluid management causes fluid overload (FO) that is associated with poor outcomes such as high mortality or prolonged ventilation^3–7^. Patients with acute kidney injury (AKI) are more likely to have FO^8^, and FO accelerates AKI progression due to decreased renal blood flow because of increased venous pressure^9,10^. As such, managing FO is important to improve outcomes in critically ill patients with AKI.

Fluid therapy can be conceptualized as four phases from early illness through the resolution of sepsis: resuscitation, optimization, stabilization, and de-resuscitation phases^11,12^. While the phases are overlapping and problematic in differentiating clearly, inadequate fluid administration, as well as inadequate management of the de-resuscitation, worsens patient outcomes. Thus, it should be carefully managed during all four phases to improve patient outcomes. Notably, since the Surviving Sepsis Guidelines highlighted a minimum of 20–30 ml/kg of fluid bolus prior to vasopressor initiation improved the mortality in patients with sepsis^1^, fluid resuscitation in the resuscitation phase is currently assumed as the gold standard therapy in sepsis. However, a recent review also reported that the amount of fluid administered did not contribute to improved survival in sepsis^13^. Thus, there is still room to investigate fluid therapy even in the early phase of critical illness.

A previous study in patients with AKI reported that increased cumulative fluid balance during the first 3 days of intensive care unit (ICU) admission was associated with higher mortality^14^; another study in critically ill patients identified that the increased fluid balance during the 24 to 48 h after ICU admission as the risk factor for mortality^4^. In addition, an investigation in ICU patients reported that higher FO on day 3 of ICU admission was associated with longer ventilator days^15^. These reports demonstrated that higher FO has an impact on poor outcomes in critically ill patients including AKI. However, since the previous studies investigated the time interval of every 24 h, the detailed timing from when FO is associated with the outcome is unclear. Aggressive fluid management is performed during the early phase after ICU admission and the timepoints that FO associated with outcomes could be earlier than previously reported; recognition of this association may contribute to changing clinical practice in fluid management and improving patient outcomes.

In this study, we hypothesized that FO could have an impact on outcomes from an earlier phase than previously reported in critically ill patients with AKI. To demonstrate our hypothesis, we analyzed the associations between FO and mortality in AKI patients using hourly data after ICU admission. In addition, since pulmonary edema can be the most considerable complication of FO as it directly inhibits oxygen uptake from the early phase of critical illness^12^, we also analyzed the associations between FO and ventilator-free days (VFDs).

## Methods

### Study setting and patients

This single-center retrospective observational study was conducted in the medical/surgical ICU of Chiba University Hospital between January 2011 and December 2015^16^. Adult (≥ 18 years old) patients, who were diagnosed with AKI within 24 h of ICU admission and stayed in the ICU for ≥ 72 h were screened for eligibility. Since AKI and end-stage renal disease (ESRD) have different prognoses even among kidney dysfunction/failure patients^17^, patients were excluded if they were diagnosed with ESRD. Patients who had missing data on body weight were also excluded. First admission data were analyzed if patients had been admitted to the ICU multiple times.

### Ethical approval

The Chiba University Hospital Certified Clinical Research Review Board approved this study (No. 3413) and because of the retrospective study design, written informed consent was waived by the Research Review Board. Opt-out on the study was posted at the entrance of our ICU to inform about the study. The study was carried out according to Ethical Guidelines for Medical and Health Research Involving Human Subjects in Japan.

### Data collection and definition

Baseline characteristics comprising age, sex, body weight, comorbidities (chronic kidney disease [CKD], hypertension [HT], diabetes mellitus [DM]), severity scores (Acute Physiology and Chronic Health Evaluation [APACHE] II score, Sequential Organ Failure Assessment [SOFA] score), and etiologies of AKI (sepsis, cardiovascular disease, hypovolemia, severe acute pancreatitis, hepatic failure, burn, major surgery, drug-induced, urinary tract obstruction, and others) were retrieved^16^. To estimate AKI and FO, hourly urine output/total fluid input/total fluid output for the first 72 h of ICU admission, serum creatinine levels at baseline and on day 1, or use of renal replacement therapy on day 1 of ICU admission were retrieved. The baseline creatinine levels were defined as the lowest documented level within 3 months to 1 week before ICU admission ^18^. In patients with missing baseline serum creatinine levels, we calculated the values according to the revised estimated glomerular filtration rate (GFR) from serum creatinine levels adapted for the Japanese population^19^, assuming an estimated GFR of 75 mL/min/1.73 m^2^^20^. In-hospital 90-day mortality and 28-day VFDs were estimated as outcome values. VFDs was defined as days patients alive without mechanical ventilation according to a previous report^21^.

AKI was diagnosed based on creatinine and urine output criteria as detailed in the Kidney Disease Improving Global Outcomes (KDIGO), (creatinine criteria, changes in serum creatinine levels from baseline to day 1 or need for renal replacement therapy (RRT); urine output criteria, urine output within 24 h after the ICU admission)^20^. When the AKI stages differed between the two criteria, we deemed the patient to be of the higher AKI stage.

Hourly fluid balance was calculated as the total fluid input (including oral intakes, intravenous and intraarterial fluids, enteral nutrition, and blood products) minus total fluid output (including urine output, ultrafiltration volume, output from surgical drains, and effusions). Cumulative %FO was calculated hourly using the following formula: (cumulative fluid balance until the time of evaluation/baseline body weight) *100 (%)^14^.

### Statistical analysis

The primary outcome was in-hospital 90-day mortality. The secondary outcome was days alive and 28-day VFDs. To clarify from what hour the cumulative %FO associates with outcomes, we performed logistic and linear regression analyses hourly from ICU admission up to 72 h and calculated each hour's odds ratio (OR)/coefficient (95% confidence interval [CI]). Multivariate regression analyses including age, sex, APACHE II score, and sepsis etiology were performed to adjust the baseline differences for both mortality and VFDs. Pearson’s chi-square test was used to analyze categorical values while the Mann–Whitney U test was used to analyze continuous values.

We also performed hierarchical clustering analysis on the cumulative %FO data to visualize the association between the different transitions of %FO and mortality. The Ward’s method of linkage, which minimizes the variance of distance between the clusters was chosen as the hierarchical clustering method^22^. The Euclidean distance was used to measure dissimilarity between the temporal trajectory of cumulative %FO^23^. The dendrogram was visualized using the Scipy library, and the optimal number of clusters was determined using the elbow method **(**See Additional Fig. 1). The resulting clusters were then characterized by calculating the median and quantiles of cumulative %FO and mortality in each cluster.

Data are expressed as median (interquartile range [IQR]) for continuous values and absolute number (%) for categorical values. Two-tailed *p*-values < 0.05 were considered statistically significant. Analyses were performed using SPSS software version 26.0.0 (IBM Corporation, Armonk, NY, USA). Python 3.8.10 packages (Scikit-learn 1.2.0, Pandas 1.3.5, NumPy 1.21.6, Scipy 1.7.3, and Matplotlib 3.2.2) were used to perform the clustering method.

## Results

### Baseline characteristics and clinical outcomes

In total, 8715 patients who were admitted to the ICU during the study period were screened. Of these, 1120 adult AKI patients were analyzed **(**See Additional Fig. 2). Non-survivors (n = 297) had significantly higher severity scores (APACHE II and SOFA scores), a higher proportion of stage 3 AKI, and increased cumulative fluid balances compared to the survivors (*P* < 0.001) **(**Table 1**)**. In addition, VFDs were significantly shorter in non-survivors than in survivors (non-survivor vs. survivors, 2 [0–12] vs 24 [0–28] days, *P* < 0.001).

**Table 1:** Baseline characteristics and clinical outcomes in acute kidney injury patients.

	Survivor (n = 903)	Non-survivor (n = 217)	*P*-value
Characteristics			
Age, years	68 (56–76)	69 (60–77)	0.17
Male sex, n (%)	571 (63.2)	148 (68.2)	0.15
Body weight, kg	61.2 (52.6–71.5)	60.0 (51.6–71.7)	0.43
Comorbidity, n (%)			
CKD	155 (17.2)	44 (20.3)	0.28
Hypertension	436 (48.3)	83 (38.2)	0.008
Diabetes mellitus	218 (24.1)	50 (23.0)	0.73
APACHE II score	23 (17–30)	32 (26–40)	< 0.001
SOFA score	7 (4–10)	11 (8–14)	< 0.001
AKI etiology, n (%)			
Cardiovascular disease	340 (37.7)	59 (27.2)	0.004
Sepsis	199 (22.0)	77 (35.5)	< 0.001
Major surgery	106 (11.8)	19 (8.8)	0.21
Hypovolemia	75 (8.3)	12 (5.5)	0.17
Severe acute pancreatitis	32 (3.5)	5 (2.3)	0.36
Hepatic failure	19 (2.1)	13 (6.0)	0.002
Urinary tract obstruction	7 (0.8)	1 (0.5)	0.62
Drug-induced	4 (0.4)	2 (0.9)	0.39
Burn	2 (0.2)	0 (0.0)	0.49
Other and unknown	115 (12.8)	29 (13.4)	0.81
KDIGO AKI, n (%)			
Stage 1	278 (30.8)	32 (14.7)	< 0.001
Stage 2	310 (34.3)	51 (23.5)	0.002
Stage 3	315 (34.9)	134 (61.8)	< 0.001
Cumulative fluid balance			
0–24 h	1248.6 (182.0 to 2831.9)	1955.0 (702.2 to 4520.5)	< 0.001
24–48 h	490.0 (− 473.0 to 1386.5)	941.3 (− 27.4 to 2098.0)	< 0.001
48–72 h	32.0 (− 800.0 to 827.5)	605.8 (− 462.4 to 1439.0)	< 0.001
Outcome			
Ventilator-free days	24 (20–28)	2 (0–12)	< 0.001

CKD, chronic kidney disease; APACHE, Acute Physiology and Chronic Health Evaluation; SOFA, Sequential Organ Failure Assessment; AKI, acute kidney injury; KDIGO, Kidney Disease Improving Global Outcome; ICU, intensive care unit.

Data are presented as a median and interquartile range for continuous variables. *P-*values were calculated using Pearson’s chi-square test or the Mann–Whitney U test.

### The association between the outcomes and fluid accumulation

**Figure 1:**
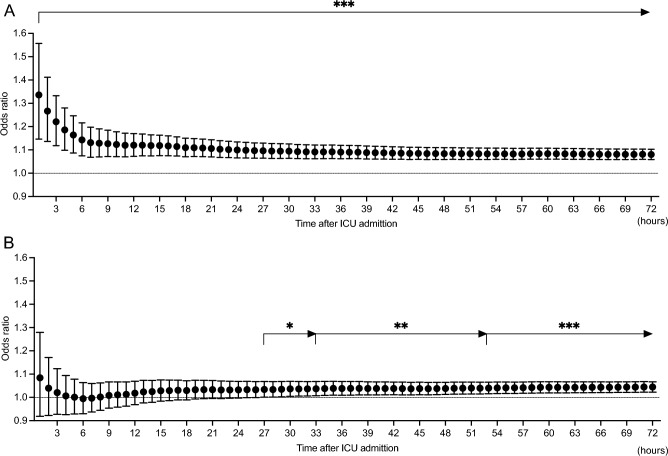
Association between percent fluid overload and in-hospital 90-day mortality in patients with acute kidney injury. (**A**) Univariate logistic regression analysis. Percent fluid overload was significantly associated with mortality from the 1st hour of intensive care unit admission. (**B**) Multivariate logistic regression analysis adjusted with age, sex, APACHE II score, and sepsis etiology. Percent fluid overload was significantly associated with mortality from the 27th hour of intensive care unit admission. Percent fluid overloads were calculated as (fluid balance until the time of evaluation/baseline body weight) *100. *: *P* < 0.05, **: *P* < 0.01, ***: *P* < 0.001.

Univariate logistic regression analysis for the association between cumulative %FO evaluated hourly from the ICU admission and in-hospital 90-day mortality demonstrated that a higher %FO was significantly associated with higher mortality from the first hour from admission, and the significance lasted up to 72 h (1st hour, OR 1.34, 95% CI 1.15–1.56, *P* < 0.001; 72nd hour, OR 1.08, 95% CI 1.06–1.10, *P* < 0.001) (Fig. 1A). Multivariate analysis adjusted for age, sex, APACHE II score, and etiology of sepsis demonstrated that a higher %FO was significantly associated with higher mortality from 27 h and the significance lasted up to 72 h (27th hour, adjusted OR 1.03, 95% CI 1.00–1.07, *P* = 0.046; 72nd hour, adjusted OR 1.04, 95% CI 1.02–1.07, *P* < 0.001) (Fig. 1B).

**Figure 2:**
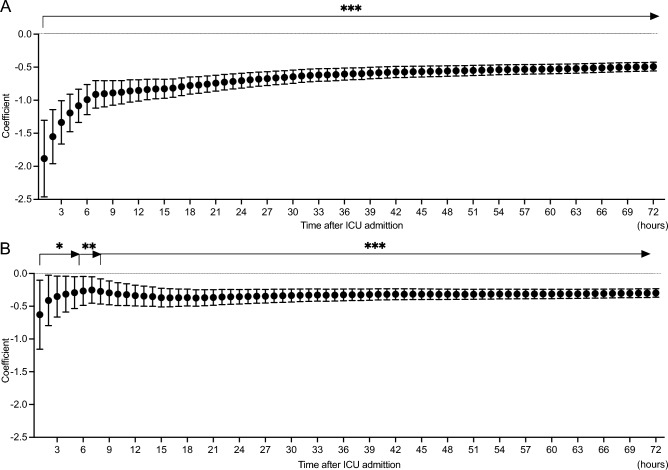
Association between percent fluid overload and ventilator-free days in patients with acute kidney injury. (**A**) Univariate logistic regression analysis. Percent fluid overload was significantly associated with ventilator-free days from the 1st hour of intensive care unit admission. (**B**) Multivariate logistic regression analysis adjusted with age, sex, APACHE II score, and sepsis etiology. Percent fluid overload was significantly associated with ventilator-free days from the 1st hour of intensive care unit admission. Percent fluid overloads were calculated as (fluid balance until the time of evaluation/baseline body weight) *100. *: *P* < 0.05, **: *P* < 0.01, ***: *P* < 0.001.

Both univariate and multivariate linear regression analysis for the association between cumulative %FO and VFDs demonstrated that a higher %FO was significantly associated with shorter VFDs from the first hour of ICU admission, lasting for up to 72 h (univariate, 1st hour, coefficient − 1.88, 95% CI − 2.46 to − 1.30, *P* < 0.001, 72nd hour, coefficient − 0.49, 95% CI − 0.56 to − 0.42, *P* < 0.001; multivariate, 1st hour, coefficient − 0.63, 95% CI − 1.16 to − 0.10, *P* = 0.019; 72nd hour, coefficient − 0.30, 95% CI − 0.36 to − 0.23, *P* < 0.001) (Fig. 2A and B).

### Clustering based on fluid accumulation

**Figure 3:**
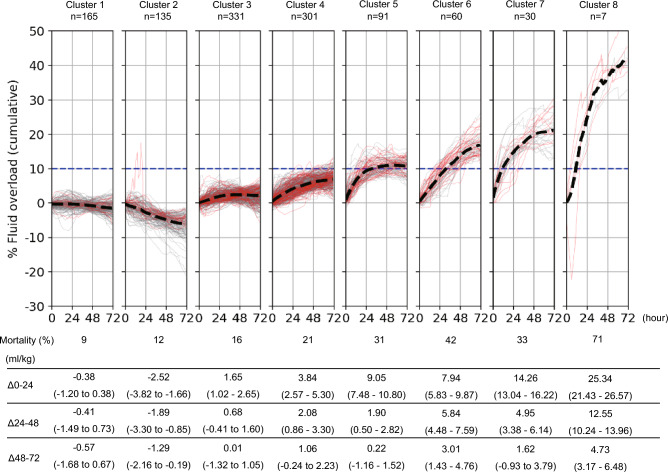
Clustering of patients with acute kidney injury according to the percent fluid overload. Patients were divided into eight groups according to the transitions of similar percent fluid overload using hierarchical clustering. Δ0-24, Δ24-48, and Δ48-72 are the changes in the percent fluid overload during the timepoints and expressed as median (interquartile range).

The patients were divided into eight groups based on %FO transition (Fig. 3). We found that the clusters with negative %FO at 72 h had low mortality rates (clusters 1 and 2), whereas the cluster with > 10% of %FO at 72 h had high mortality rates (clusters 5–8). The greater the increase in %FO during hours 0 to 24, the more mortality was worsened, with the exception of cluster 6. Cluster 6 had the 2nd highest mortality among clusters, but the %FO during hours 0–24 was lower than that in clusters 5 and 7, which had lower mortalities than did this cluster. While the association between %FO during hours 0–24 and mortality was inconsistent in cluster 6, a relatively higher %FO during hours 24 to 48 and 48 to 72 was observed in this cluster.

## Discussion

The present study demonstrated that increased cumulative %FO was associated with higher mortality and shorter VFDs from the early phase of critical illness; notably, the cumulative %FO was significantly associated with mortality from the 27th hour of ICU admission and the association between %FO and VFDs had significance from the first hour of ICU admission and lasting for up to 72 h. Clustering analysis by %FO transition illustrated that the increased %FO in the first 24 h and an exceeding %FO of > 10% at 72 h was associated with high mortality.

Previous studies reported that the increased cumulative fluid balance on day 3 was associated with higher mortality in critically ill patients with AKI or who received invasive mechanical ventilation^14,15^; investigation in septic patients suggested that fluid balance during hours 24 to 48 after ICU admission was associated with hospital mortality whereas there was no association in the first 24 h^4^. Our analysis of the association between hourly FO data and mortality suggested that the exact timepoints of FO should be earlier than that of previous reports, and it should be considered in the fluid resuscitation management of critically ill patients.

Edema causes tissue damage via oxygen and metabolite diffusion, distorted tissue architecture, obstruction of capillary blood flow/lymphatic drainage, and disturbed cell-to-cell interactions^24^. FO exacerbates organ edema which impairs organ functions^24,25^. Pulmonary edema leads to impaired gas exchange and hypoxemia, which makes a requirement for ventilatory support^26^. A previous investigation in patients who received mechanical ventilation reported that increased fluid balance on day 3 was significantly associated with shortened VFDs^15^, which is consistent with our results. A noteworthy finding of the present study is that the %FO was associated with VFDs from the first hour of ICU admission. This indicates that physicians need to pay attention to controlling fluid balance as much as possible even in the initial phase of treatment.

In using hierarchical clustering analysis, we found that a negative %FO during the first 24 h was associated with decreased mortality, whereas mortality was increased as %FO was higher in the positive range. Added to the association of mortality with fluid balance at this period, a larger increase of FO in the following period was associated with increased mortality. In addition, we confirmed that a cumulative %FO > 10% at 72 h was associated with higher mortality. A previous study of patients who received invasive mechanical ventilation reported that the patients who could achieve to have a negative fluid balance on day 3 by deresuscitative measures had lower mortality than the patients who had positive fluid balance^15^. In addition, a previous study in patients with AKI demonstrated that the patients who had cumulative %FO > 10% during the first 3 days of treatment had high mortality of 49.1%^14^. In this study, mortality in clusters 5–8, where the patients had %FO > 10%, was 31–71% (Fig. 3). Our results are consistent with those of previous reports, and our clustering analysis with visualization may contribute to clarifying the association.

Our study has several strengths. Notably, we monitored the FO hourly, which contributed to finding the detailed timing from when the FO was associated with outcomes. In addition, we selected patients broadly. Thus, our results can be interpreted as a general phenomenon. Furthermore, our clustering analysis identified the groups that had different trends of fluid accumulation and mortality, which can help to consider the patients’ prognosis in clinical practice. However, this study also has several limitations. First, since this is a single-center retrospective study, it may have selection bias. However, we screened more than 8000 patients and included over 1000 patients in this study, which may contribute to generalization of the patients and reducing the bias. Also, we couldn’t evaluate the fluid balance before ICU admission. It may have an impact on the transition of fluid administration after ICU admission. In addition, while a previous report suggested that the FO leads the organ dysfunction^27^, we can’t identify the causal relationship between the FO and outcomes. The disease severity may be associated with the necessity of fluid administration; the ability to respond to de-resuscitation can be caused by patients’ recovery. In terms of phases of fluid therapy, our results may be affected by the patient's progress during the critical illness. Even though, multivariate analysis with severity score revealed a significant association between FO and mortality or VFDs from earlier timing than previous reports. Second, we could not differentiate the type of administered fluid. Crystalloids or colloids may have different roles and result in different outcomes^28,29^. Third, we did not consider the administration of diuretic agents. Further study is required to observe the impact of diuretic agents on the outcomes.

In the conclusions**,** a higher %FO was associated with increased mortality and shortened VFDs. These associations were seen from an earlier phase than previously reported. Fluid balance should be administered with a physiological target or goal in place in critically ill patients with AKI, even during the initial treatment phase.

### Supplementary Information

Supplementary Figures.

## Data Availability

The datasets used and/or analyzed during the current study are available from the corresponding author upon reasonable request.

## Abbreviations

AKI: Acute kidney injury
APACHE: Acute Physiology and Chronic Health Evaluation
CI: Confidence interval
CKD: Chronic kidney disease
DM: Diabetes mellitus
FO: Fluid overload
GFR: Glomerular filtration rate
HT: Hypertension
ICU: Intensive care unit
IQR: Interquartile range
KDIGO: Kidney Disease Improving Global Outcomes
OR: Odds ratio
RRT: Renal replacement therapy
SOFA: Sequential Organ Failure Assessment
VFDs: Ventilator-free days
