# Supplementary material for: Associations between fluid overload and outcomes in critically ill patients with acute kidney injury: a retrospective observational study
Source: Sci Rep. 2023 Oct 13;13:17410. doi: 10.1038/s41598-023-44778-0 (PMC10575912; doi:10.1038/s41598-023-44778-0)
Supplement: Supplementary file 1 — Supplementary Figures. [file 41598_2023_44778_MOESM1_ESM.docx]

**Additional Fig 1. Hierarchical clustering dendrogram and distortion**.

A. Hierarchical clustering dendrogram.

B. Distortion for the elbow method.

**Additional Fig 2.** Patient flowchart.

AKI, acute kidney injury; BW, body weight; ESRD, end-stage renal disease; ICU, intensive care unit
